# Suspended Lead Suits and Radiation Exposure in Interventional Echocardiographers

**DOI:** 10.1001/jamanetworkopen.2025.58134

**Published:** 2026-03-11

**Authors:** David A. McNamara, Jeffrey M. Decker, Michael W. McNamara, Mohamad A. Kenaan, David M. Cameron, Stacie VanOosterhout, Jessica L. Parker, Ryan D. Madder

**Affiliations:** 1 Frederik Meijer Heart & Vascular Institute, Corewell Health West, Grand Rapids, Michigan; 2 Corewell Health Research Institute, Corewell Health, Grand Rapids, Michigan; 3 Department of Cardiovascular Medicine, William Beaumont University Hospital, Corewell Health East, Royal Oak, Michigan

## Abstract

**Importance:**

Percutaneous left atrial appendage occlusion (LAAO) is frequently performed with an interventional echocardiographer (IE) in close proximity to the patient and radiation source, increasing the risk for occupational radiation exposure. Yet optimal radiation shielding methods for IEs remain unknown.

**Objective:**

To assess whether using a suspended lead suit reduces IE radiation dose compared with traditional lead apron shielding during LAAO procedures.

**Design, Setting, and Participants:**

In this blinded cross-sectional study, IE radiation exposure data were prospectively collected during all LAAO procedures conducted for adults at a single quaternary care center from February 21 to August 22, 2023. Head-level dosimeters were worn by IEs who were using suspended lead suits during sequential LAAO procedures conducted at the center.

**Exposures:**

Use of a suspended lead suit or traditional lead apron radiation protection device.

**Main Outcomes and Measures:**

The primary outcome was measured personal dose equivalents at head level to IEs per case using real-time radiation dosimeters. Dosimeter-derived physician radiation doses and procedural radiation (dose area product) were compared with a control group of 30 sequential historical LAAO cases performed with IEs wearing traditional lead aprons from July 1, 2016, to January 31, 2018.

**Results:**

Overall, 125 patients (mean [SD] age, 78 [8] years; 77 [61.6%] male) were included. Among 95 cases in which IEs used suspended lead suits, the median (IQR) radiation dose was 0.0 (0.0-0.3) μSv, which was significantly lower than 30 cases in which IEs wore traditional lead aprons (median [IQR], 10.6 [5.8-24.1] μSv; *P* < .001). Radiation doses were undetectable in 60% of IEs (57of 95) using suspended lead suits compared with 0% of IEs (0 of 30) using traditional lead aprons (*P* < .001). Radiation doses of at least 20 μSv were observed in 0 of 95 cases (0%) with suspended lead suits vs 9 of 30 cases (30%) with traditional lead aprons (*P* < .001). Similar associations were noted in exploratory analyses adjusting IE radiation doses for procedural dose area product (mean [IQR], 0.0 [0.0-0.0] μSv/Gy × cm^2^) for suspended lead suits vs traditional lead aprons (mean [IQR], 0.6 [0.3-1.0] μSv/Gy × cm^2^; *P* < .001).

**Conclusions and Relevance:**

In this cross-sectional study, the use of suspended lead suits was associated with large reductions in head-level radiation doses to IEs compared with the use of traditional lead aprons during LAAO cases. These findings have important ramifications for the risk of occupational radiation exposure to IEs and potential shielding mechanisms during structural heart interventions.

## Key Points

**Question:**

Does using a suspended lead suit reduce interventional echocardiographer radiation exposure compared with using traditional lead apron shielding during left atrial appendage occlusion procedures?

**Findings:**

In this cross-sectional study investigating radiation exposure to interventional echocardiographers during 125 cases, the use of suspended lead suits was associated with significant reductions in head-level radiation doses to interventional echocardiographers compared with those using traditional lead aprons, achieving a median radiation dose of 0.0 μSv (vs 10.6 μSv with traditional lead aprons).

**Meaning:**

Findings of this study suggest that use of suspended lead suits may offer substantial reductions in the occupational radiation exposure to interventional echocardiographers during structural heart interventions.

## Teaser

This cross-sectional study assesses whether using suspended lead suits reduces interventional echocardiographer radiation exposure compared with using traditional lead apron shielding during left atrial appendage occlusion procedures.

## Introduction

During fluoroscopically guided structural heart procedures, interventional echocardiographers (IE) are in close proximity to the patient, the principal source of scatter radiation. Long-term exposure to scatter radiation in the cardiac catheterization laboratory has been associated with multiple adverse health effects among interventional cardiologists, including premature cataract formation,^1,2^ early carotid atherosclerosis,^3^ and possibly left-sided brain malignant lesions.^4^ Data have suggested that IEs are at elevated risk for radiation exposure and receive even higher doses compared with their interventional cardiologist colleagues during the same procedures.^5^ Although multiple technologies intended to protect interventional cardiologists from scatter radiation have been developed, few interventions have been investigated for IEs. Given the rapid growth in the field of interventional echocardiography, interventions to reduce the occupational risk of IE are warranted to help inform mitigation efforts.

Suspended lead suits, composed of a 1.0-mm lead apron and a 0.5-mm lead equivalent transparent head and neck shield, are supported overhead to eliminate weight on the operator. Use of suspended lead suits are associated with marked (>90%) reductions in radiation exposure to interventional cardiologists during diagnostic coronary angiography and coronary interventions.^6,7^ The role of suspended lead suits for IEs, however, remains unclear, particularly in light of the frequent transesophageal echocardiography (TEE) probe manipulations and c-arm angles used during structural heart interventions. Herein, we present a pragmatic, single-center, investigator-led cross-sectional study evaluating occupational radiation exposure to IEs using suspended lead suits compared with traditional lead aprons during left atrial appendage occlusion (LAAO) cases. We hypothesized that the use of suspended lead suits by IEs would be associated with lower occupational radiation doses compared with controls using traditional lead apron shielding.

## Methods

### Study Population

This single-center, investigator-initiated cross-sectional study was designed to prospectively investigate occupational radiation doses to IEs during LAAO procedures. The study was conceived, designed, and conducted by investigators at Corewell Health without input from industry. The institutional review board at Corewell Health West Butterworth Hospital, Grand Rapids, Michigan, approved the protocol. IEs consented to participation in the study, and patients consented to Health Insurance Portability and Accountability Act authorization to access their medical records for research purposes. This study followed the Strengthening the Reporting of Observational Studies in Epidemiology (STROBE) reporting guideline.

Radiation exposure data were prospectively collected during all LAAO cases during which IEs performed TEE at a single quaternary care center. Exclusion criteria included cases in which TEE guidance was not planned or used or in which IEs were not wearing dosimeters during part or all of the LAAO case; fluoroscopy system–derived radiation metrics were not available; the suspended lead suit was not used for a portion of the case; a procedure concurrent with LAAO was performed in the same setting; the LAAO procedure was aborted part way through; or the patient was 18 years of age or younger. All LAAO procedures involved implantation of a commercially available closure device (Watchman, Boston Scientific or Amulet, Abbott). Procedures were performed using a transseptal approach and were guided by fluoroscopy and TEE as directed by the performing physician. No procedures included intracardiac echocardiography.

IEs were positioned at the head of the patient as outlined previously, and fluoroscopy and cine were used according to operator discretion.^5^ State-of-the-art fluoroscopy systems (AlluraClarity; Philips) with real-time image noise reduction technology (Clarity IQ; Philips) were used. TEE imaging in all LAAO cases was conducted either by, or with direct supervision of, board-certified cardiologists with expertise in image-guided structural heart interventions. In 15 of 95 LAAO cases in which suspended lead suits were used, a cardiology fellow was the primary TEE probe operator and as such used the suspended lead suit and wore the dosimetry badge as described in the next section. Zero of 30 operators in the traditional lead shielding group were fellows. Data on race and ethnicity were not collected as a part of this study.

### Radiation Protection

#### Intervention Group, Suspended Lead Suits

IEs used a suspended lead suit (Zero-Gravity; Biotronik) consisting of a 1.0-mm-thick suspended lead body and a 0.5-mm lead equivalent acrylic face shield meant to provide face, head, and neck protection. Data for the suspended lead suit group were obtained from February 21 to August 22, 2023. A lead thyroid collar was worn for this study solely for the purpose of attaching the radiation dosimeter in a standardized location to reduce measurement variability. Additionally, a mobile, height-adjustable, accessory lead shield was available for positioning between the patient and IE for additional protection. This supplemental, portable lead shield, (the use of which was at the discretion of the IE) has a fixed lower half and a height-adjustable upper half with a lead equivalency of 0.5 mm. The upper portion of this shield could be raised when the TEE probe was not being manipulated and could be lowered during probe manipulation at the IE’s discretion. The protective measures and room set-up are depicted in eFigure 1 in Supplement 1.

#### Control Group, Traditional Lead Aprons

Prospective head-level radiation doses were collected using dosimeters (RaySafe i2; GE Health Care) in an identical location as in the suspended lead suit group for IEs during 30 consecutive LAAO cases. These data were obtained at the same institution from July 1, 2016, to January 31, 2018, as previously described.^5^ These control group data were included to provide direct comparison for the aforementioned prospective dosimeter data. Radiation protection in the control group consisted of a traditional lead skirt, apron, and thyroid collar. Suspended lead suits were not used in the control group. The height-adjustable accessory lead shield described in the previous section was used in an identical manner in the control group. Identical fluoroscopy systems and procedural rooms were used, and the radiation badge location was identical to facilitate between-group comparisons.

### Primary Outcome

Head-level IE radiation dose was the primary outcome of interest and was analyzed on a per case basis. IE radiation doses were the personal dose equivalent at a depth of 10 mm in tissue collected using a commercially available real-time dosimetry system (RaySafe i3; GE Health Care). This dose is referred to as the physician radiation dose throughout the manuscript. To measure head-level radiation dose, each physician wore a dosimeter located on the left anterior side of the thyroid collar facing the radiation source as previously described, external to any leaded covering.^5,8^

### Patient and Procedural Data

#### Patient-Level and Procedural Data

Age, sex, height, weight, body mass index (calculated as weight in kilograms divided by height in meters squared), body surface area, and relevant medical history were obtained from the medical record. Procedural radiation metrics recorded for each case included the fluoroscopy time, air kerma (AK), and dose area product (DAP), which were automatically calculated by the fluoroscopy imaging system. Both AK and DAP are commonly used metrics to estimate patient radiation dose; AK is defined as the radiation delivered to air at a reference point located 15 cm on the x-ray tube side of isocenter, and DAP is defined as the product of AK and the x-ray field area.^9^

#### Participant-Level Data

Deidentified participant-level IE data were recorded. These data included the number of years in practice (since completion of fellowship) at the time of study initiation and the height of the IEs. These data were obtained by interviewing the physician directly as well as by querying quality and outcomes databases.

### Statistical Analysis

Data analyses were conducted between October 1, 2023, and December 14, 2024. Descriptive statistics were used to summarize baseline characteristics and outcome measures. Normally distributed continuous variables are given as the mean (SD). Nonnormally distributed continuous variables are reported as the median (IQR). Categorical variables are reported as number (percentage). Normality was assessed by visual inspection of histogram plots, the Shapiro-Wilk *P* value, and skewness and kurtosis values between −2 and 2. *P* values for comparison of continuous variables between the suspended lead suit group and the traditional lead apron control group were derived from 2-sample independent *t* tests when data were normally distributed or from the Mann-Whitney rank test when data were not normally distributed. Exploratory subgroup analyses based on the LAAO device used were conducted. *P* values for comparison of categorical variables were generated with χ^2^ analysis or a Fisher exact test when the expected cell counts were below 5 in more than 20% of the cells. A 2-sided *P* < .05 was considered statistically significant. No data were imputed if missing. To obtain a 95% power to detect an 82% reduction in radiation exposure compared with traditional lead apron aprons, 90 cases with a suspended lead suit were necessary.^6,7^ Factoring in a 20% risk of exclusion or screen failure, an estimated 108 individuals were needed to be screened for enrollment. A total of 119 patient cases were screened for inclusion with 24 exclusions with the study workflow, outlined in eFigure 2 in Supplement 1.

To compare IE radiation doses between groups, 2 methods were used: (1) unadjusted dosimeter-derived radiation doses were reported; and (2) IE radiation doses were normalized to DAP. Normalizing to DAP facilitates accounting for differences in procedural radiation use in the 2 groups, similar to prior work.^10^ In addition to reporting the personal dose equivalent per case, the frequency of a physician radiation dose of 0.0 μSv per case and at least 20 μSv per case were reported. The 20 μSv per case dose was selected as it is roughly 10-fold higher than the mean physician radiation dose received by interventional cardiologists across a variety of procedures and has been reported in previous studies.^5,11^ IE participants and study organizers were blinded to the radiation doses until after dosimeter data collection were completed. All statistical analyses and figures were generated using SAS, version 7.1 (SAS Institute Inc).

## Results

### Study Population

In total, 119 LAAO cases were performed in which the IE used a suspended lead suit for radiation protection. Of these cases, 95 met criteria for study inclusion (eFigure 2 in Supplement 1). An additional 30 consecutive LAAO cases using traditional lead aprons for radiation protection were included as a control group for comparison. Baseline characteristics of both patient groups are presented in Table 1. Overall, 125 study patients had a mean (SD) age of 78 (8) years, were predominantly male (48 [38.4%] female, 77 [61.6%] male), and often had obesity (mean [SD] body mass index, calculated as weight in kilograms divided by height in meters squared, 30.1 [5.9]). A high cardiovascular risk factor burden was noted in both groups. No significant differences in patient-level variables were identified between cases with suspended lead aprons and those with traditional lead aprons, except for hypertension, which was more common in the suspended lead suit group (23 [76.7%] vs 87 [91.6%]). Six IEs performed the LAAO procedures. They had been in practice a mean (SD) of 6.6 (4.5) years following completion of fellowship training and were a mean (SD) height of 175.3 (7.6) cm.

**Table 1.** Baseline Characteristics of Patients Who Underwent Left Atrial Appendage Occlusion, Stratified by Radiation Shielding Method

Variable	Patients, No. (%)		
	Overall (n = 125)	Traditional lead apron (n = 30)	Suspended lead suit (n = 95)
Age, mean (SD), y	78 (8)	80 (6)	77 (8)
Sex			
Female	48 (38.4)	13 (43.3)	35 (36.8)
Male	77 (61.6)	17 (56.7)	60 (63.2)
BMI, mean (SD)	30.1 (5.9)	30.7 (5.8)	30.0 (6.0)
Hypertension	110 (88.0)	23 (76.7)	87 (91.6)
Diabetes	31 (24.8)	5 (16.7)	26 (27.4)
History of CAD	66 (52.8)	17 (56.7)	49 (51.6)
History of stroke	27 (21.6)	10 (33.3)	17 (17.9)

Abbreviations: BMI, body mass index (calculated as weight in kilograms divided by height in meters squared); CAD, coronary artery disease.

### Procedural Radiation Dose Metrics

Procedural radiation characteristics are outlined in Table 2, with an overall median (IQR) fluoroscopy time of 7.0 (4.7-9.6) minutes, AK of 81 (40-153) mGy, and DAP of 8.1 (3.6-19.6) Gy × cm^2^. Significantly lower fluoroscopic time, AK, and DAP were observed in cases using suspended lead suits when compared with those using traditional lead aprons.

**Table 2.** Procedural Radiation Metrics and Interventional Echocardiographer Radiation Dose During Percutaneous Left Atrial Appendage Occlusion, Stratified by Shielding Method

Variable	Shielding method, median (IQR)			*P* value^a^
	Overall (n = 125)	Traditional lead apron (n = 30)	Suspended lead suit (n = 95)	
Radiation metric				
Fluoroscopy time, min	7.0 (4.7-9.6)	9.3 (6.9-14.2)	6.5 (4.2-9.2)	.001
Air kerma, mGy	81 (40-153)	164 (97-268)	62 (33-119)	<.001
DAP, Gy × cm^2^	8.1 (3.6-19.6)	22.6 (12.9-28.8)	5.5 (2.9-11.8)	<.001
Radiation dose to interventional echocardiographer				
Dose, μSv	0.1 (0.0-1.6)	10.6 (5.8-24.1)	0.0 (0.0-0.3)	<.001
Dose normalized to DAP, μSv/Gy × cm^2^	0.0 (0.0-0.1)	0.6 (0.3-1.0)	0.0 (0.0-0.0)	<.001

Abbreviation: DAP, dose area product.

^a^ Analyzed using the Mann-Whitney rank test.

### Interventional Echocardiographer Radiation Doses

**Figure 1.**
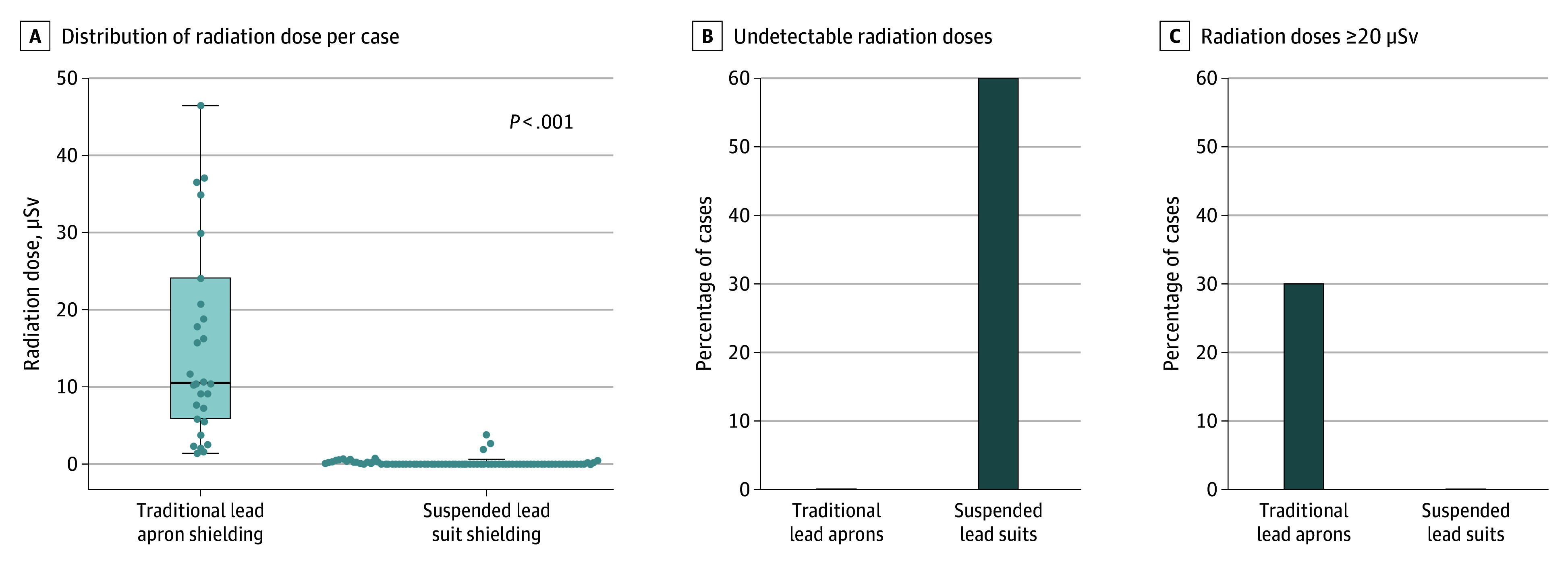
Box Plot and Bar Graphs of Dosimeter-Derived Head-Level Radiation Doses to Interventional Echocardiographers During Fluoroscopically Guided Left Atrial Appendage Occlusion Extreme outliers were removed in (A) for graphical purposes. Boxes represent interquartile range; line in the center of the box, median; error bars, minimum and maximum values not including outliers. Graphical depiction of cases with undetectable radiation doses (B) and with doses of at least 20 μSv per case (C). Group with suspended lead suit shielding, n = 95; with traditional lead apron shielding, n = 30.

**Figure 2.**
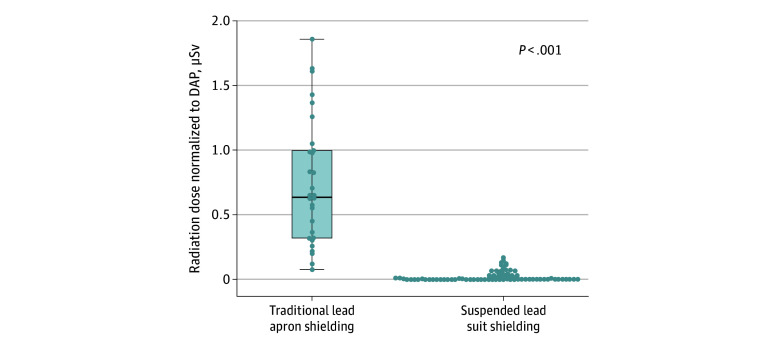
Box and Whisker Plot Showing Radiation Doses to Interventional Echocardiographers Indexed to the Dose Area Product (DAP) During Fluoroscopically Guided Left Atrial Appendage Occlusion Group with suspended lead suit shielding, n = 95; with traditional lead apron shielding, n = 30.

For the 95 cases using a suspended lead suit, the median (IQR) IE radiation dose was 0.0 (0.0-0.3) μSv. In comparison, the median (IQR) IE radiation dose using traditional lead aprons was 10.6 (5.8-24.1) μSv (Table 2). These results are displayed graphically in Figure 1. To account for differences in the amount of procedural radiation used between groups, physician radiation doses were normalized to DAP, similar to prior work.^10^ The normalized radiation doses are shown in Table 2 and Figure 2. The median (IQR) normalized IE radiation dose was significantly lower among cases using suspended lead suits compared with conventional lead aprons (0.0 [0.0-0.0] μSv/Gy × cm^2^ vs 0.6 [0.3-1.0] μSv/Gy × cm^2^; *P* < .001).

When analyzed categorically, cases using suspended lead suit shielding demonstrated lower rates of radiation exposure compared with traditional lead apron shielding across multiple clinically relevant thresholds. We found that 60% (57 of 95) of the suspended lead suit group had undetectable head level radiation doses compared with 0% (0 of 30) of the traditional lead shielding group (*P* < .001). Additionally, 0 of 95 cases (0%) using suspended lead suit shielding reached thresholds of at least 20 μSv per case. In contrast, cases using traditional lead aprons demonstrated a higher incidence of occupational radiation of at least 20 μSv per case, with 30.0% of LAAO cases (9 of 30) reaching this threshold (*P* < .001) (Figure 1). Additionally, there was little variation in radiation exposure in cases using suspended lead suits (median [IQR], 0.0 [0.0-0.3] μSv), whereas larger variance was noted in cases during which traditional lead aprons were used (median [IQR], 10.6 [5.8-2.4] μSv).

### Subgroup Analyses

In the suspended lead suit group, 27 cases implanted the Amulet LAAO device, and 68 cases implanted the Watchman LAAO device. When analyses were stratified by the 2 LAAO devices, the cases in which Amulet devices were implanted required more fluoroscopic imaging time and had more procedural radiation used (Table 3). Notably, however, this did not translate into differences in radiation exposure to IEs during the Amulet and Watchman procedures (median [IQR], 0.0 [0.0-0.3] μSv vs 0.0 [0.0-0.2] μSv; *P* = .62). Similarly, exploratory analyses of cases using suspended lead suits demonstrated lower procedural radiation use (both AK and DAP) in the 5 cases in which no portable lead shield was used compared with those in which a portable lead shield was used (eTable in Supplement 1). However, no differences in physician radiation doses were observed between cases using a portable lead shield and those without the portable shield.

**Table 3.** Radiation Metrics and Dosimeter-Derived Head-Level Radiation During Percutaneous Left Atrial Appendage Occlusion Cases Using Suspended Lead Suit Shielding, Stratified by Device Implanted

Radiation metric	Device implanted, median (IQR)		*P* value^a^
	Amulet (n = 27)	Watchman (n = 68)	
Fluoroscopy time, min	8.0 (6.5-9.3)	6.0 (3.9-8.8)	.003
Air kerma, mGy	82 (38-149)	57 (31-107)	.13
DAP, Gy × cm^2^	8.3 (3.6-19.7)	5.2 (2.2-9.7)	.02
Physician radiation dose, μSv	0.0 (0.0-0.3)	0.0 (0.0-0.2)	.62

Abbreviation: DAP, dose area product.

^a^ Analyzed using Mann-Whitney rank sum test.

## Discussion

The primary result of this study is that the use of suspended lead suits by IEs during LAAO cases was associated with marked reductions in occupational radiation doses to IEs compared with the use of traditional lead aprons. In comparison with the median IE radiation dose of 10.6 μSv for traditional lead aprons, the use of suspended lead suits had a median radiation dose of 0.0 μSv. Although significant differences in procedural radiation use were noted between groups, the observed differences in IE radiation doses remained significant after normalization to DAP. Remarkably, IEs received no detectable head-level radiation in 60% of cases with suspended lead suits, whereas with traditional lead aprons, head-level radiation exposure was detectable in every case. Furthermore, when using traditional lead aprons, head-level radiation doses to IEs of at least 20 μSv were commonplace and observed in nearly one-third of cases (30.0%), but in contrast were not observed in any of the 95 LAAO cases when using suspended lead suits. These findings have important occupational health implications for the growing structural heart team, in particular the IE community who are exposed to high radiation doses compared with their interventional cardiology counterparts during structural heart interventions.^5,12^ Although the results of the present study are encouraging, future implementation and cost-effectiveness studies are needed to determine the most appropriate method to mitigate occupational radiation exposure to members of the structural heart team.

The past several years have experienced substantial growth in the study of occupational radiation exposure in the catheterization laboratory, especially among interventional cardiologists exposed to occupational radiation doses during coronary angiography and percutaneous revascularization. However, to date, there are only small single-center studies focusing on radiation exposure to IEs,^5,12^ and until recently IE radiation exposure has not been an area of focus for cardiology societies. Further compounding this issue, there has been rapid growth in the number of structural heart procedures performed throughout the US and worldwide, with a corresponding influx of IEs in the cardiac catheterization laboratory. Analyses recently noted substantial institutional variation in radiation use during transcatheter valve interventions, indicating IEs at some centers may be at particularly high risk for receiving excessive radiation doses during structural heart interventions.^13^

Recent experience has identified that radiation use is modifiable, as demonstrated by large state-wide quality improvement interventions during coronary angiography and percutaneous coronary revascularization.^14,15^ However, there are limited investigations to date into interventions to mitigate radiation exposure to IEs during structural heart procedures. In one of the only studies focusing on radiation-reducing technology for IEs, Crowhurst et al,^12^ describe IE radiation doses in a small, single-center investigation after implementing a ceiling-mounted lead shield in 50 cases. Implementing the shield was associated with a 5-fold reduction in radiation exposure to the IE during a broad range of structural heart cases. While an important step forward, the generalizability of the results by Crowhurst et al to other structural heart cases including LAAOs remains unclear, in part as radiation exposure differs based on the procedure performed. The exclusive focus of the present study on LAAO cases is of particular importance to reduce procedure-related variability (eg, differences in projection angles). LAAO was selected as a worst-case scenario for IE radiation exposure based on the high amount of steep c-arm angulation throughout the procedure. Additionally, the continual TEE probe adjustments required during LAAO procedures can make the use of portable lead shields, such as those studied by Crowhurst et al,^12^ intermittent and challenging in contrast to the suspended suit in the present study. To our knowledge, no other interventions to reduce radiation exposure during structural heart interventions beyond those by Crowhurst et al^12^ have been studied in the past 7 years. Furthermore, the present investigation represents the largest comparison of shielding techniques for IEs to date and focuses on a specific procedure to reduce procedure-related variation.

In the present study, the reduction in radiation exposure to IEs was marked, with a median head-level radiation dose with suspended lead suits of 0.0 (IQR, 0.0-0.3) μSv. To put these results in context, the radiation dose to IEs using suspended lead suits in this study was well below the dose reported in prior work in which a ceiling-mounted shield was used (median, 0.5 [IQR, 0.0-1.4] μSv).^12^ The radiation doses to IEs using suspended lead suits remained several-fold lower than doses to cardiologists performing right heart catheterization, endomyocardial biopsy, diagnostic coronary angiography, and percutaneous coronary intervention while wearing traditional lead aprons.^10^ Importantly, the low radiation exposure to IEs did not depend on the device implanted, despite more fluoroscopic imaging time and higher radiation production during cases using the Amulet compared with the Watchman devices.

Beyond the large absolute and relative reduction in median IE radiation dose was the low level of variability observed during cases in which suspended lead suits were used. Undetectable radiation exposure to the IE was present in 60% of cases in the present study. Absent were the wide variance and frequent high doses (≥20 μSv) observed among cases using traditional lead aprons. The significant reduction in dose variability and frequent cases with high radiation exposure is clinically meaningful to IEs but may be of particular emphasis to female IEs, who are at elevated risk during childbearing years.^16^

Putting the issue of radiation protection for IEs into context, a recent international survey noted poor radiation protection for IEs.^17^ Importantly, the primary method of shielding used by greater than 90% of IEs was lead aprons and thyroid shields, which was the very comparator assessed in this study. Therefore, the intervention of suspended lead suits in the present study compared with traditional lead apron shielding may potentially help to fill an important knowledge gap about optimizing radioprotective shielding. Our findings also highlight an important unmet public health need: the health and protection of IEs. To this end, recent societal efforts, including a white paper from the American Society of Echocardiography^18^ and programing from the Society for Cardiovascular Angiography & Interventions, have highlighted the importance of this issue. Additional advocacy for radiation protection to IEs remains a priority, and research into the optimal shielding techniques and implementation for IEs during structural heart cases should be a focus of future work.

### Limitations

This study has limitations. First, it focused on LAAO cases at a single institution. As a result, generalization of the results to other procedures should be done with caution. While some cases included supervised fellows in training, we viewed this as a strength that improves the generalizability of the study. Second, although it is possible concomitant improvements in radiation safety culture may have been implemented, the magnitude of radiation reduction observed in this study is far greater than expected from radiation safety culture practices observed in the coronary realm.^6,7,14^ Additional multicenter studies will be important to explore site-to-site variation in radiation doses to IEs. Importantly, there were no doses of at least 20 μSv per case in the group using suspended lead suits and very few nonzero values (Figure 1A) in this group. Thus, the reduction in radiation exposure likely depends very little on the user of suspended lead suits. While the dosimeter detection level used in the present study is a common methodology used in prior studies,^5,6,7,8,10^ there remains disagreement in how the lower values should be handled. Additionally, while this study did not investigate the cost difference or ergonomic benefits between traditional lead aprons and suspended lead suits, both certainly play important roles in implementation.^19,20^ Further studies focusing on the cost ramifications of radiation shielding for IEs are suggested to explore the cost-effectiveness of implementing this protection for health care systems.

## Conclusions

In this cross-sectional study, the use of suspended lead suits was associated with large reductions in head-level radiation doses to IEs compared with the use of traditional lead aprons. These data are of public health importance and may help educate appropriate protective approaches for IEs, particularly given the rapid rise in transcatheter structural heart interventions throughout the US.

## Notes

Supplement 1. **eFigure 1.** Catheterization Laboratory Setup - Interventional Echocardiographer, Interventional Cardiologist, and Sonographer Position **eFigure 2.** Study Flow Diagram Left Atrial Appendage Occlusion Cases Utilizing Suspended Lead Suit Shielding **eTable.** Radiation Metrics and Dosimeter Data Stratified by the Use of a Portable Lead Shield

Supplement 2.
Data Sharing Statement
