# Supplementary material for: Suspended Lead Suits and Radiation Exposure in Interventional Echocardiographers
Source: JAMA Netw Open. 2026 Mar 11;9(3):e2558134. doi: 10.1001/jamanetworkopen.2025.58134 (PMC12980244; doi:10.1001/jamanetworkopen.2025.58134)
Supplement: Supplement 1. — eFigure 1. Catheterization Laboratory Setup - Interventional Echocardiographer, Interventional Cardiologist, and Sonographer Position eFigure 2. Study Flow Diagram Left Atrial Appendage Occlusion Cases Utilizing Suspended Lead Suit Shielding eTable. Radiation Metrics and Dosimeter Data Stratified by the Use of a Portable Lead Shield [file jamanetwopen-e2558134-s001.pdf]

Supplementary Online Content

McNamara DA, Decker JM, McNamara MW, et al. Suspended lead suits and radiation exposure in interventional echocardiographers. *JAMA Netw Open*. 2026;9(3):e2558134. doi:10.1001/jamanetworkopen.2025.58134

eFigure 1. Catheterization Laboratory Setup - Interventional Echocardiographer, Interventional Cardiologist, and Sonographer Position

eFigure 2. Study Flow Diagram Left Atrial Appendage Occlusion Cases Utilizing Suspended Lead Suit Shielding

eTable. Radiation Metrics and Dosimeter Data Stratified by the Use of a Portable Lead Shield

This supplementary material has been provided by the authors to give readers additional information about their work.

© 2026 McNamara DA et al. *JAMA Network Open*.

eFigure 1. Catheterization Laboratory Setup - Interventional Echocardiographer, Interventional Cardiologist, and Sonographer Position

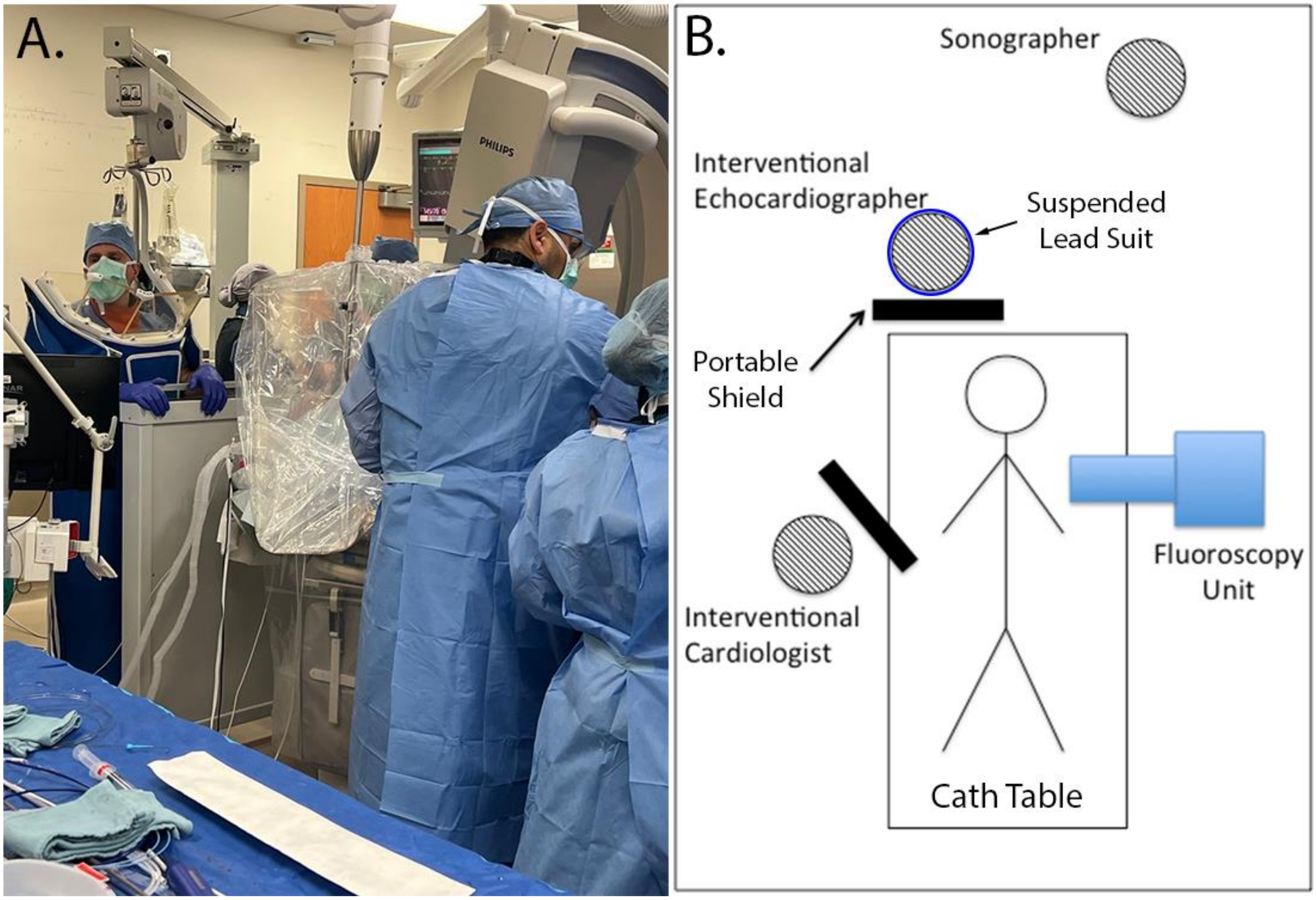

(**A**) Left atrial appendage occlusion cases were performed with an interventional echocardiographer standing at the patient's head to manipulate the transesophageal echocardiogram probe throughout the procedure. The interventional echocardiographer used a suspended lead suit (as depicted in the image) or traditional lead aprons (not depicted) as described elsewhere in the text. In addition, interventional echocardiographers in both groups used identical mobile, height-adjustable, accessory lead shielding. The upper section of the shield was raised to a height that allowed the interventional echocardiographer to extend their

© 2026 McNamara DA et al. *JAMA Network Open*.

arms over the shield (retracted/down in this image) to manipulate the transesophageal echocardiogram probe throughout the case. **B**) The overhead diagram shows the relative position of the interventional echocardiographer, interventional cardiologist, and sonographer to the patient and fluoroscopy unit. The suspended lead suit described above is labelled and depicted with a blue ring. The mobile accessory lead shield described above is labelled as “Portable Shield.”

© 2026 McNamara DA et al. *JAMA Network Open*.

eFigure 2. Study Flow Diagram Left Atrial Appendage Occlusion Cases Utilizing Suspended Lead Suit Shielding

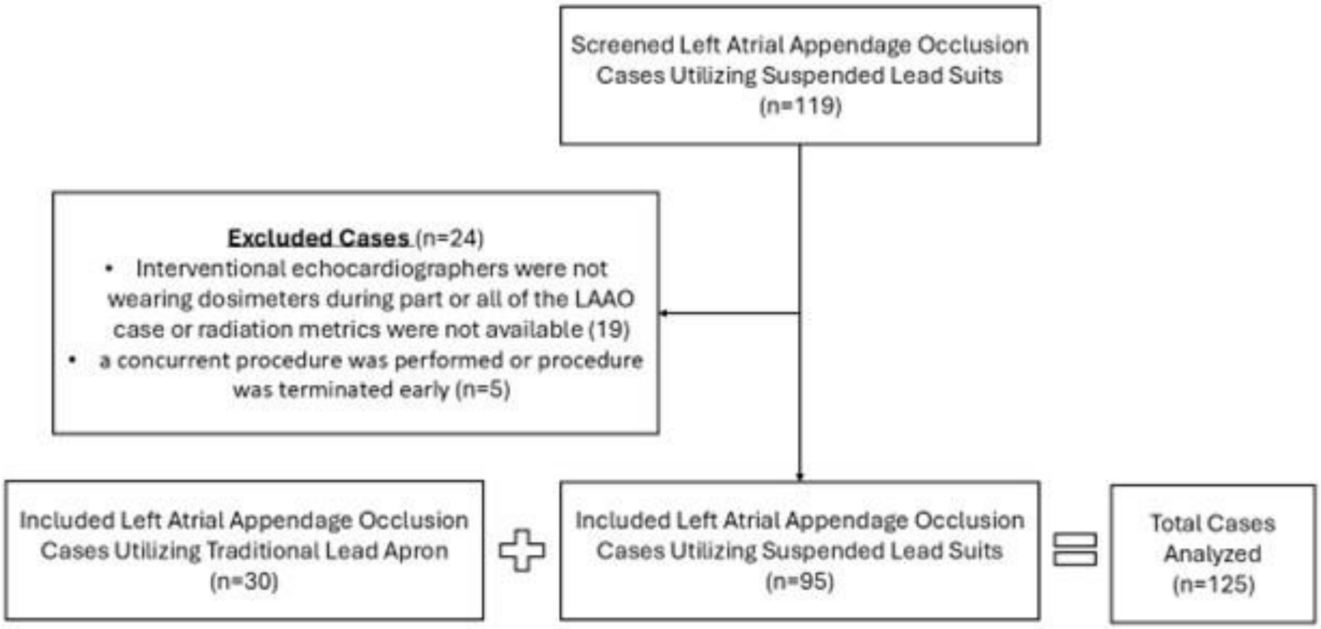

© 2026 McNamara DA et al. *JAMA Network Open*.

eTable. Radiation Metrics and Dosimeter Data Stratified by the Use of a Portable Lead Shield

	No MD Portable Lead Shield (N=5)	MD Portable Lead Shield (N=90)
Fluoroscopy time, mins	6.5 [2.7, 6.7]	6.7 [4.3, 9.2]
Air Kerma, mGy	37 [35, 53]	66 [33, 120]
DAP, mGy x cm^2	2.5 [2.3, 2.9]	5.6 [3.3, 11.8]
Physician radiation dose, μSv	0.0 [0.0, 0.0]	0.0 [0.0, 0.3]

Abbreviations are the same as Table 1 in the main article.

© 2026 McNamara DA et al. *JAMA Network Open*.
