# Supplementary material for: Suspended Lead Suits and Radiation Exposure in Interventional Echocardiographers
Source: JAMA Netw Open. 2026 Mar 11;9(3):e2558134. doi: 10.1001/jamanetworkopen.2025.58134 (PMC12980244; doi:10.1001/jamanetworkopen.2025.58134)
Supplement: Supplement 2. — Data Sharing Statement [file jamanetwopen-e2558134-s002.pdf]

Data Sharing Statement

McNamara. Suspended Lead Suits and Radiation Exposure in Interventional Echocardiographers. *JAMA Netw Open*. Published March 11, 2026. doi:10.1001/jamanetworkopen.2025.58134

Data
Data available: No
